# The Non-Pathogenic *Fusarium oxysporum* Fo47 Induces Distinct Responses in Two Closely Related Solanaceae Plants against the Pathogen *Verticillium dahliae*

**DOI:** 10.3390/jof7050344

**Published:** 2021-04-28

**Authors:** Javier Veloso, José Díaz

**Affiliations:** 1 Grupo de Investigación de Fisioloxía e Aplicacións das Plantas (FISAPLANT), Departamento de Bioloxía, Facultade de Ciencias, Universidade da Coruña, Campus da Zapateira, 15071 A Coruña, Spain; jose.diaz.varela@udc.es; 2 Centro de Investigaciones Científicas Avanzadas (CICA), Campus de Elviña, 15008 A Coruña, Spain

**Keywords:** *Fusarium oxysporum*, Fo47, biocontrol, induce resistance, ethylene, *Capsicum annuum*, *Solanum lycopersicum*

## Abstract

The non-pathogenic *Fusarium oxysporum* Fo47 is able to protect *Capsicum annuum* (pepper) but not in *Solanum lycopersicum* (tomato) against the pathogen *Verticillium dahliae*. Transcriptomics of the plant during the interaction with Fo47 shows the induction of distinct set of genes in pepper and tomato. The number of differentially expressed (DE) genes in pepper (231 DE genes) is greater than the number of DE genes in tomato (39 DE genes) at 2 days after the treatment with Fo47. Ethylene related genes were present among the DE genes in both plants, and the up-regulation of ethylene biosynthetic genes was observed to be triggered during the interaction of both plants with Fo47. The treatment with MCP (1-Methylcyclopropene, an ethylene-competitive inhibitor) reduced the Fo47 protection in pepper against *Verticillium dahliae*. Intriguingly, Fo47 was able to protect the ethylene-insensitive tomato mutant Never-ripe (Nr) against *Verticillium dahliae*, but not the tomato wilt type cv Pearson. Overall, ethylene is shown to be an important player in the response to Fo47, but its role depends on the host species.

## 1. Introduction

*Fusarium oxysporum* Fo47 has previously been demonstrated to protect several plant species from pathogenic *Fusarium* strains [1,2,3,4,5]. The mechanism triggered in plants treated with Fo47 is not well understood, but it involves antagonistic interactions with the pathogen and the induction of defences in the plant. In tomato, treatment with Fo47 increased levels of PR proteins in the roots and leaves, but at lower levels than a pathogenic strain *F. oxysporum* f. sp. *lycopersici* [6,7]. A non-pathogenic *Fusarium solani* was also able to protect tomato plants against the root pathogen *Fusarium oxysporum* f. sp. *radicis-lycopersici*, and to elicit induced systemic resistance against the tomato foliar pathogen *Septoria lycopersici*. This non-pathogenic *Fusarium* did not induce PR genes in the root or in the leaves. Moreover, mutant tomato lines Never-ripe (*Nr*) and epinastic (*epi1*), both impaired in ethylene-mediated plant responses, treated with this non-pathogenic *Fusarium* were not protected against the subsequent inoculation with the pathogenic *F. oxysporum*, suggesting an involvement of ethylene (ET) in the protection obtained by the application of the non-pathogenic *Fusarium* [8]. Recently, a study using tomato mutants impaired in the ethylene (ET), salicylic acid (SA), and jasmonic acid (JA) signaling/biosynthetic pathway showed no reduction in the protection conferred by Fo47 against the pathogenic *Fusarium oxysporum* f. sp. *lycopersici* [9]. However, in pepper, HPLC measurements after treatment with Fo47 showed increases in 12-oxo-phytodienoic acid (OPDA), salicylic acid, and jasmonyl isoleucine [10]. This points to an intricate network of hormones that might have independent branches. The response to the different hormones also depends on the plathosytem [11]. For instance, SA is involved in resistance against *Botrytis cinerea* in tomato but not in tobacco [12], showing the adaptability/plasticity of the immune system to that specific interaction, changing not only with the host but also with the different pathogen species or even strains of the same species [11].

The simplified common idea is the existence of two antagonistic signaling pathways, the SA- and JA/ET-signaling pathways [13]. However, the response of tomato to the pathogen *V. dahliae* involves the interplay between these plant hormones [14]. The R-gene mediated response caused by the interaction of *V. dahliae* with *Ve1*-carrying tomato depends on the components of the SA-pathway [15]. The tomato cultivar Pearson used in this work carries no *Ve1* gene, and *Capsicum annuum Ve1* homologs have been only identified in silico [16]. Fo47 increased the levels of SA in the roots of pepper cv Padron 56 h after Fo47 treatment [10]. Fo47 did also increase the levels of two compounds (12-oxo-phytodienoic acid and jasmonyl-isoleucine) related to JA-signaling in pepper [10]. After inoculation with *V. dahliae*, only 12-oxo-phytodienoic acid was increased in the Fo47-treated plants [10]. Dhar et al. [14] proposed that both SA and JA signaling play a role in defense against Verticillium sp.; the SA-mediated response appears to take a predominant role during the initial biotrophic phase, while JA-mediated response restricts the damages from the systemic pathogen spread during the necrotrophic phase. Fo47 enhances these responses by inducing SA- and JA-related compounds.

Ethylene has been involved in both resistance and susceptibility to Verticillium [17]. Robison et al. [17] showed that post-infection ethylene enhances Verticillium wilt development in tomato, whereas its presence at the time of infection inhibits disease development. In pepper, expression of a basic *PR-1* is positively regulated by ET [18]. Combinations of ET with SA or JA reduce the induction exerted by ET alone. Strikingly, a combination of SA and JA induces this basic *PR-1* even more than ET alone [18]. In tobacco, basic isoforms of *PR-1* are also activated by ET [19]. In tomato, basic *PR-1* proteins are activated by both SA and ET precursors, as well as by tobacco mosaic virus [20]. Transcriptomic assays determined that the gene expression profile induced by ET and JA overlapped only 50% of the genes [21].

The response triggered by non-pathogenic *Fusarium* is not well established, and further investigation is needed before any comparison with other models can be established. Fo47 is able to reduce the symptoms of *V. dahliae* in pepper cv Padron, but not in tomato cv Pearson at the inoculum concentrations used here. To further understand the mechanism orchestrating the response to the biocontrol agent Fo47, an expression profile has been carried out in pepper and tomato treated with this beneficial fungus. The expression profile in both plants showed the presence of several ethylene related genes. An inhibitor and a mutant of ethylene perception was used in pepper and tomato, respectively, to assay the involvement of ethylene in the Fo47-induced response (FIR).

## 2. Materials and Methods

### 2.1. Plant Material and Treatment Method

Seeds of *Capsicum annuum* cv Padron (pepper) were stored at 4 °C. The pepper seeds were disinfected prior to usage by incubation in 10% (*v*/*v*) commercial bleach for 10 min and then washed and soaked overnight in distilled water before being sown in sterile vermiculite. Seeds of *Solanum lycopersicum* cv Pearson (tomato) and the mutant Never-ripe (Nr) were sterilized and dried in clean-bench prior storage at 4 °C. Tomato seeds were sown in sterile vermiculite. The *Solanum lycopersicum* mutant Never-ripe (Nr) was sown, grown, and inoculated similarly to Pearson. Plants were grown in a growth chamber at 25 °C with a photoperiod of 16 h light and 8 h darkness. Tomato plants were used for the treatment with Fo47 20 days after sowing and pepper plants were treated with Fo47 30 days after sowing.

*F. oxysporum* Fo47 was kindly provided by Claude Alabouvette and Christian Steinberg (UMR INRA, Dijon). The *Fusarium* treatment of the plants was performed according to Díaz et al. [22] with some modifications. Fo47 inoculum was obtained from cultures growing in potato dextrose broth medium for 7 days. The culture was filtered and the filtrate was centrifuged at 2500× *g* for 5 min. The pellet was resuspended in sterile distilled water and the concentration was adjusted to 10^6^ conidia per ml. The roots of the pepper or tomato were dipped into the conidial suspension of Fo47 for 3 h. The control plants were treated with sterile distilled water instead of Fo47 conidia. Some pepper plants were exposed to 1-methylcyclopropene (MCP), an inhibitor of ethylene perception, in a sealed container [23] at a final concentration of 0.2 μL L^−1^. A control group of plants was kept in a container with no chemical added. Containers were opened after 4 h of treatment, and following aeration, the plants were then treated with Fo47 as described above.

### 2.2. Pathogen Material and Inoculum Preparation

The *V. dahliae* isolate UDC53Vd was previously obtained by our research group in Galicia (Northwest of Spain) from an diseased pepper plant collected from a farm [24,25]. *V. dahliae* was grown in potato dextrose agar (PDA) plates for 3 weeks. The inoculum was obtained by flooding the plates with 10 mL of sterile distilled water and gently rubbing the plate with a glass Drigalski spatula to liberate the conidia [22]. The concentration was adjusted to 10^6^ or 2 × 10^6^ conidia per ml for pepper or tomato, respectively. Inoculum concentration was optimized experimentaly for pepper and tomato to obtain readeable symptoms with the lowest inoculum concentration. UDC53Vd is a pepper isolate and requires lower concentrations in pepper. For tomato, 10^6^ *V. dahliae* conidia per ml did not produce consistent visible symptoms. An assay using 2 × 10^6^ conidia for Fo47 and 2 × 10^6^ conida for *V. dahliae* was also performed, with similar results.

### 2.3. Inoculation with Verticillium dahliae

After the treatment with water or Fo47, the plants were placed in sterile flasks with nutrient solution and incubated for 48 h in a growth chamber. Then, the plant roots were placed into the *V. dahliae* inoculum for 45 min or 2 h for pepper or tomato, respectively. Incubation time was optimized experimentally for pepper and tomato to obtain readable symptoms with the shortest incubation time. UDC53Vd is a pepper isolate and requires shorter incubation time in pepper. A challenge control group treated with sterile water instead of *V. dahliae* was also prepared. Afterwards, the plants were transplanted into pots containing a sterile 4:1 (*v/v*) mixture of soil and perlite and placed following a Latin square design in a culture chamber at 25 °C day/18 °C night, with a 16-h photoperiod. The stem length, the number of wilt leaves, and the fresh and dry weight were recorded four weeks or three weeks for pepper or tomato, respectively. Three independent experiments were carried out for pepper (8 plants per treatment and experiment, each plant was considered a replication *n* = 192) and two independent experiments for tomato (8 plants per treatment and experiment, each plant was considered a replication *n* = 128).

### 2.4. Transcriptomic Profiling

Samples of tomato and pepper stems were collected 48 h after Fo47 treatment without pathogen inoculation. In all cases, the samples (5 plants per sample) were frozen in liquid nitrogen and stored at −80 °C. Total RNA was extracted from the homogenized samples as described in the protocol of the BioRad Aurum^TM^ Total RNA Mini kit. Total RNA integrity was evaluated by microfluidic analysis using the Agilent 2100 Bioanalyzer with an RNA LabChip^®^ Kit. The RIN (RNA integrity number) was always higher than 9. Total RNA samples were prepared following the protocol of the Affymetrix GeneChip^®^ 3′ IVT Express Kit. In the GeneChip 3′ IVT Express Protocol, total RNA undergoes reverse transcription to synthesize first-strand cDNA. This cDNA is then converted into a double-stranded DNA template for transcription. In vitro transcription synthesizes aRNA and incorporates a biotin-conjugated nucleotide. The aRNA is then purified to remove unincorporated NTPs, salts, enzymes, and inorganic phosphate. Fragmentation of the biotin-labeled aRNA prepares the sample for hybridization onto GeneChip 3′ expression arrays. This process was experimentally validated using TaqMan^®^ RT-PCR [26].

The protocol of the GeneChip Hybridization, Wash, and Stain Kit was followed to hybridize the fragmented aRNA into the Affymetrix GeneChip Tomato Genome Array. GeneChips were hybridized in an Affymetrix GeneChip Hybridization Oven 645 for 16 h. Washing and staining were performed in an Affymetrix GeneChip Fluidics Station 450. GeneChip scanning was carried out in the GeneChip Scanner 3000 7G. GeneChip data quality control, background correction, normalization, and summarization methods were carried out with Affymetrix Expression Console™ Software. The GeneChip Tomato Genome Array contains 10,038 tomato probe sets + 11 tomato control probe sets. Only probes with a 100% match in the tomato and pepper transcriptomes were considered for the analysis, retaining 82% of the probes in the array. All control probes had a perfect match in both organisms.

The data was processed with the end-to-end workflow for differential gene expression using Affymetrix microarrays [27]. A linear model was fit to the data using the limma package. Genes were considered to be differentially expressed with an adjusted *p*-value of less than 0.05 and a log2change fold change bigger than 0.6 or smaller than −0.6. Gene ontology (GO) annotations of differentially expressed genes were assigned based on biological processing using the NetAffx™ Analysis Center.

*Arabidopsis thaliana* homologues were used to assign GO annotations when they were unavailable for tomato. *Arabidopsis thaliana* homologues were assigned to each differentially expressed gene using National Center for Biotechnology Information (NCBI) Blastx with a e-value of less than 1 × 10^−6^. They were also used for pathway reconstruction using the Database for Annotation, Visualization, and Integrated Discovery (DAVID) for Functional Annotation Bioinformatics and Microarray Analysis. DAVID matches the genes within well-described pathways in the Kyoto Encyclopedia of Genes and Genomes (KEGG) databases and retrieves a *p*-value for each pathway based on the number of genes fitting within and the proximity between them.

### 2.5. qPCR Gene Expression

Pepper and tomato stems were collected 48 h after Fo47 treatment without pathogen inoculation. In all cases, the samples (5 plants per sample) were frozen in liquid nitrogen and stored at −80 °C. Total RNA was extracted from the homogenized samples as described in the protocol of the BioRad Aurum Total RNA Mini kit. The retrotranscription was carried out following the protocol of the BioRad iScript^TM^ cDNA Synthesis Kit.

The cDNA samples were analysed with the Biorad iCycler^TM^ iQ System following the protocol described by Silvar et al. [28,29]. The assay was performed for two genes: a basic pathogenesis-related protein 1 (for pepper *CaPR1*, gene ID: 107840155; and for tomato *SlPR1*, gene ID: 100191111) and 1-aminocyclopropane-1-carboxylate oxidase (for pepper *CaACO1*, gene ID: 107853805; and for tomato *SlACO5*, gene ID: 543800). The constitutive expression of the actin gene (for pepper *CaACT*, gene ID: 107840006; and for tomato *SlACT*, gene ID: 101250165) was used for internal normalization [30].

The qPCR reactions were prepared with Biorad 1X iQ SYBR Green Supermix, 0.3 μM of each primer, and 2.5 μL of cDNA for a 50 μL end volumen reaction. The PCR program started with a 2 min denaturation step at 95 °C followed by 40 cycles of amplification (95 °C for 20 s, 58 °C for 25 s, and 72 °C for 50 s) and finished with an elongation step of 5 min at 72 °C. The data analysis was carried out with Biorad Optical System Software 3.0. The efficiency was calculated and the outcoming Ct values were processed by the Pfaffl method [30] to obtain the relative expression values.

### 2.6. Statistical Analysis

All statistical analyses were performed using R Studio. The linear model fitting from the limma package was used to analyse transcriptomic data from the Affymetrix single-channel microarrays. Percentage of wilt leaves were analysed by Mann–Whitney–Wilcoxon test (α = 0.05). The rest of the *Verticillium* inoculation experiments were analysed with a one-way ANOVA (α = 0.05) followed by Duncan tests for multiple comparisons [31]. Significant differences are reported in the text and shown in the figures.

## 3. Results

### 3.1. Fo47 Protects Pepper against V. dahliae but Not Tomato

**Figure 1:**
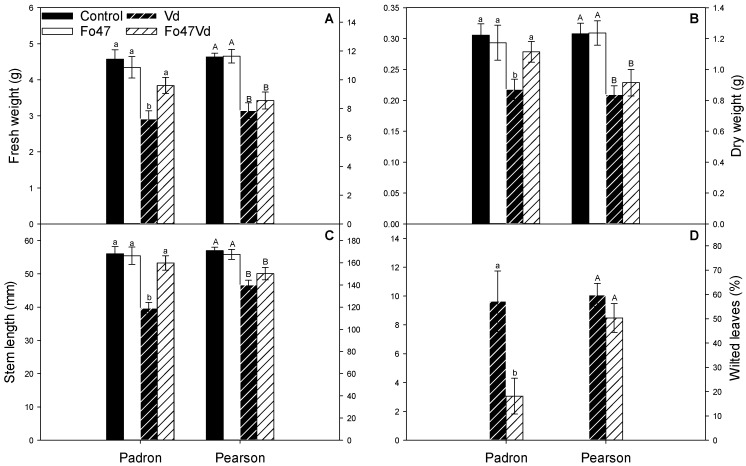
Fresh weight (**A**), dry weight (**B**), stem length (**C**), and wilted leaves (**D**) of pepper cv Padron and tomato cv Pearson. Scales for pepper are at the left side and scales for tomato are at the right side. Plants were inoculated with *Verticillium dahliae* (Vd), treated with *Fusarium oxysporum* Fo47 (Fo47), or both (Fo47Vd). Control plants were inoculated and treated with sterile distilled water (Control). Means ± the standard errors are shown. Different letters indicate significant differences in each group (Padron or Pearson) in a one-way ANOVA (α = 0.05) followed by Duncan tests (**A**–**C**) or a Mann–Whitney–Wilcoxon (α = 0.05) test (**D**). Three or two independent experiments were carried out for pepper or tomato, respectively (8 plants per treatment and experiment, each plant was considered a replication *n* = 192 or *n* = 128).

Fo47 was able to reduce the symptoms caused by *V. dahliae* in pepper cv Padron by increasing the fresh weight, dry weight, and stem length and reducing the wilted leaves of the *V. dahliae* inoculated plants (“Padron” in Figure 1). In the experiments reported here, wild type tomato cv. Pearson treated with Fo47 did not display any diminution in Verticillium wilt symptoms (“Pearson” in Figure 1).

### 3.2. Fo47 Triggers Different Transcriptome Reprogramming in Pepper and Tomato but Both Include Ethyelene-Related Genes

**Figure 2:**
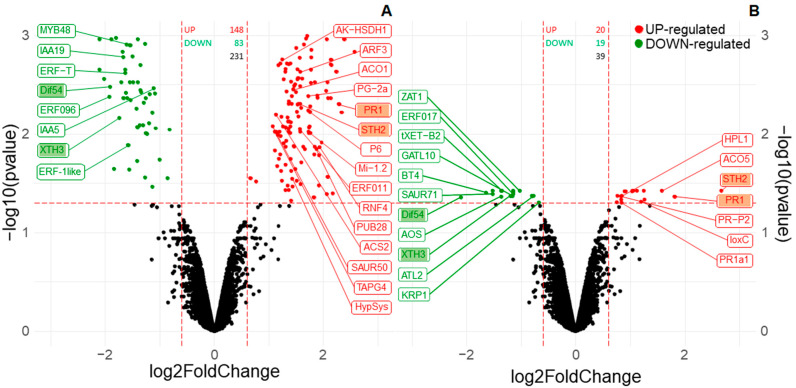
Differentially expressed (DE) genes in pepper cv Padron (**A**) and tomato cv Pearson (**B**). Red circles represent up-regulated genes and green circles represent down-regulated genes. Dashed red lines represent the cutting point for *p*-value (horizontal line) and foldchange (vertical lines). The total number of over-expressed genes is indicated in red-colored text, and down-regulated genes are indicated in green-colored text. Only biologically significant genes (GO functions or path-reconstruction) are indicated with labels.

The expression profiles of tomato cv Pearson and pepper cv Padron were analysed 48 h after treatment with Fo47. The analysis shows different genetic responses to Fo47 between both solanaceae species. In pepper, a total of 231 genes were detected to be differentially expressed (DE) after Fo47 treatment, while only 39 genes were observed in tomato (Figure 2). Among them, only four genes were differentially expressed in both species (Table 1), two up-regulated and two down-regulated. The two up-regulated genes were two pathogenesis related genes (PR1 and STH2). The two down-regulated genes were related to cellular cell wall organization, a xyloglucan endotransglucosylase (XTH3) involved in hemicellulose rearrangement, and an extensin-like protein (Dif54) that codes a hydroxyproline-rich glycoprotein that is thought to form crosslinked protein networks in the plant cell wall.

**Figure 3:**
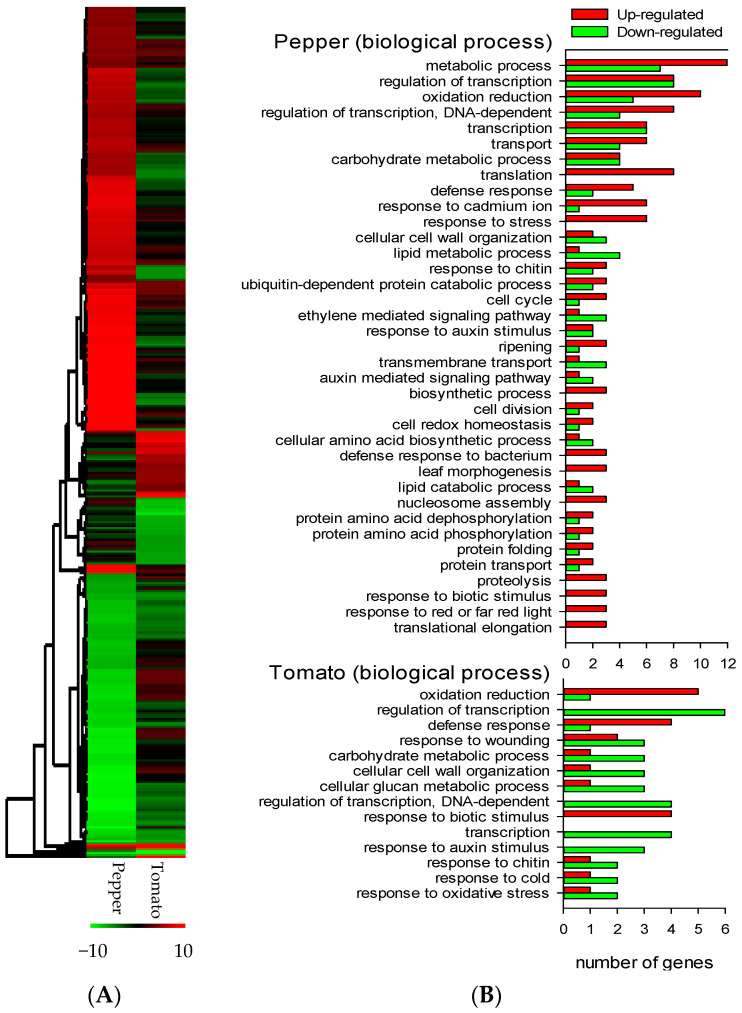
Comparison of differentially regulated genes in tomato and pepper after Fo47 treatment. Expression profile of differentially regulated genes in pepper and tomato clustered by Euclidean distance (**A**). The most relevant GO biological processes of the differentially expressed genes sorted by the number of genes in each category in pepper and tomato (**B**).

Besides these shared elements, pepper showed another 227 DE genes, while tomato showed another 35 DE genes. The heatmap shows that the expression of these genes follows different profiles in each of the species (Figure 3a). 135 genes of pepper and 29 of tomato could be sorted by their gene ontology (GO) biological process (Figure 3b).

Cellular cell wall organization is down-regulated in both solanaceous plants, while defence response is up-regulated in both plants. The defence response and the response to biotic stimuli are related in both organisms to the up-regulation of PR-proteins, *PR1a1*, *STH2*, *PR-P2*, *PR-1*, and *P6* (Table 2).

**Figure 4:**
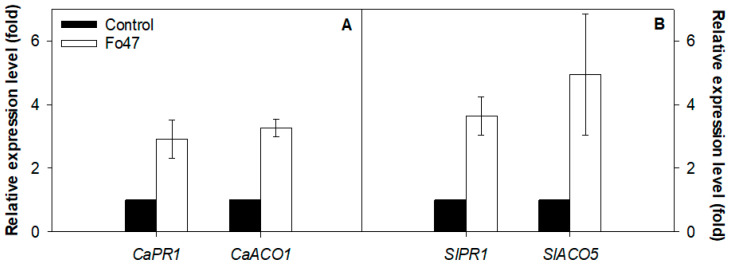
Expression of *CaPR1* and *CaACO1* in pepper (**A**) and expression of *SlPR1* and *SlACO5* in tomato (**B**). Five plants per treatment were used in 3 independent experiments. Fo47 plants were inoculated with the Fo47 strain and control plants were inoculated with sterile distilled water. Data are shown as a relative expression of the control group as described by the Pfaffl method (Pfaffl, 2001).

Ethylene is present in the response of pepper to Fo47; two genes involved in its synthesis are up-regulated and four transcription factors responsive to ethylene are also involved (Table 3). Genes related to ethylene were also differentially expressed in tomato, but to a lower extent; only a gene related to ethylene biosynthesis and an ethylene responsive transcription factor (Table 3) were differentially expressed in tomato. The ACO gene was over-expressed in pepper (ACO1) and tomato (ACO5). qPCR showed that ACO and PR1, which were also common to both solanaceous plants (Table 1), were over-expressed more than double in pepper (CaPR1, CaACO1) and tomato (SlPR1, SlACO5) after Fo47 treatment (Figure 4).

Besides ethylene related genes, genes responsive to auxin stimulus were mainly down-regulated in both solanaceous plants (Table 4). It is not clear if ethylene and auxin crosstalk might occur during Fo47-induced resistance, but auxin signaling is related to necrotrophic pathogens in *A. thaliana,* since mutants defective in auxin signaling were more susceptible to the necrotrophic fungi *Plectosphaerella cucumerina* and *B. cinerea* [32].

**Figure 5:**
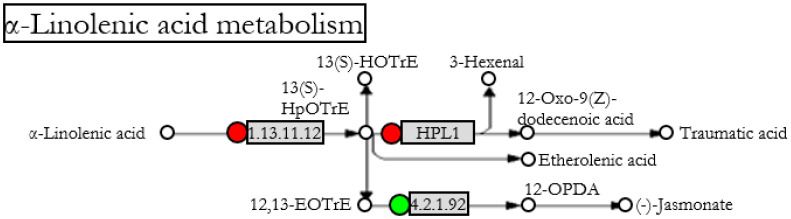
α-Linolenic acid metabolism pathway containing differentially expressed genes in tomato (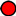: up-regulated, 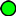: down-regulated) after Fo47 treatment. The pathway has been simplified from the original in KEGG databases (accession: SLY00592).

**Figure 6:**
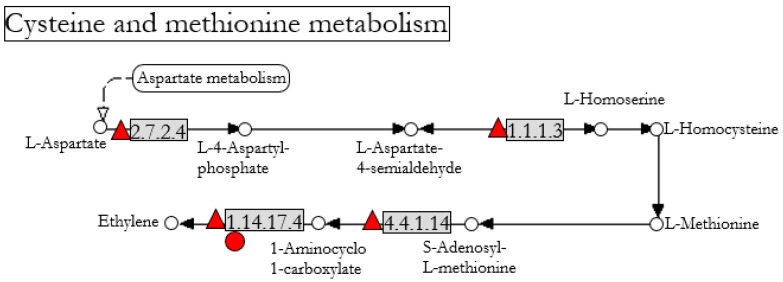
Cysteine and methionine metabolism containing differentially expressed genes in pepper (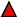: up-regulated) and tomato (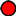: up-regulated) after Fo47 treatment. The pathway has been simplified from the original in KEGG databases (accession: CANN00270).

Using DAVID, two pathways of the KEGG databases were found to be differentially expressed in tomato and pepper during interaction with Fo47 (Figure 5, Table 5 and Figure 6, Table 6). The α-Linolenic acid metabolism pathway (KEGG pathway SLY00592, Figure 5) was significantly represented by three genes in tomato related to jasmonic acid biosynthesis: a lipoxygenase, a fatty acid hydroperoxide lyase, and an allene oxide synthase (Figure 5 and Table 5). The down-regulation of the allene oxide synthase (AOS) gene indicates that 13-hydroperoxylinolenic acid is being catabolized by hydroperoxy lyase (HPL1), forming volatile aldehydes and traumatic acid rather than forming jasmonic acid via the allene oxide synthase. Related to the α-Linolenic pathway, the biological processes’ response to wounding was also represented in tomato but not in pepper (Figure 3b). In response to wounding, the genes from the α-Linolenic pathway and transcription factors involved in response to abiotic stresses are included.

The cysteine and methionine metabolism (KEGG pathway CANN00270, Figure 6) was the second pathway identified. Three genes in pepper involved with ethylene biosynthesis represented this pathway: an aspartokinase/homoserine dehydrogenase, a 1-aminocyclopropane-1-carboxylate synthase, and a 1-aminocyclopropane-1-carboxylate oxidase (Table 6 and Figure 6). Only one gene in this pathway was up-regulated in tomato (Table 4).

### 3.3. Ethylene Role in Fo47-Induced Resistance Depends on the Host

**Figure 7:**
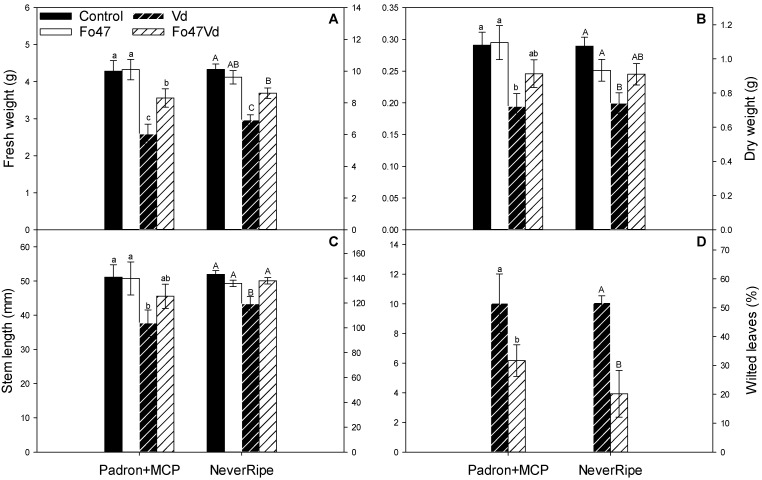
Fresh weight (**A**), dry weight (**B**), stem length (**C**), and wilted leaves (**D**) of pepper cv Padron treated with the ethylene-perception inhibitor methylcyclopropene (Padron+MCP) and the tomato ethylene-insensitive mutant Never-ripe (Never-ripe). Scales for pepper are at the left side and scales for tomato are at the right side. Plants were inoculated with *Verticillium dahliae* (Vd), treated with *Fusarium oxysporum* (Fo47), or both (Fo47Vd). Control plants were inoculated and treated with sterile distilled water (Control). Means ± the standard errors are shown. Different letters indicate significant differences in each group (Padron+MCP or Never-ripe) in a one-way ANOVA (α = 0.05) followed by Duncan tests (**A**–**C**) or a Mann–Whitney–Wilcoxon (α = 0.05) test (**D**). Three or two independent experiments were carried out for pepper or tomato, respectively (8 plants per treatment and experiment, each plant was considered a replication *n* = 192 or *n* = 128).

Pepper cv Padron treated with MCP showed a partial reduction in the Fo47-induced resistance by not significantly increasing the dry weight and stem length of the *V. dahliae* inoculated plants and increasing the wilted leaves (“Padron+MCP” in Figure 7). The tomato ethylene insensitive mutant, Never-ripe, treated with Fo47 showed increased Fo47-induced resistance compared with Pearson by increasing the fresh weight and stem length and reducing the wilted leaves of the *V. dahliae* inoculated mutant plants (“Never-ripe” in Figure 7).

## 4. Discussion

Fo47 is able to reduce the symptoms of *V. dahliae* in pepper cv Padron (Figure 1) but not in tomato cv Pearson (Figure 1) at the inoculum concentrations used here. Antagonistic interactions of Fo47 with the pathogen have been described as an important part of the resistance response observed in Fo47-treated plants [10]. Antagonistic interactions have not been ruled out in this work, and further experiments will need to be conducted to determine the importance of antagonistic interactions on the tomato/pepper dichotomy.

The induced responses observed in pepper and tomato are different depending on the plant species. Intriguingly, ET-insensitive tomato, Never-ripe, treated with Fo47 showed *V. dahliae* symptom reduction (Figure 7) under the same conditions in which the wild-type tomato cv Pearson treated with Fo47 did not show any reduction in *V. dahliae* symptoms (Figure 1). Never-ripe mutant possesses a mutation that impedes the bonding of ethylene to the ethylene receptor NR, also known as LeETR3. Therefore, NR cannot be deactivated by ET, so NR constitutively inhibits the ET-responsive genes [33]. The deactivation of the ET-responsive genes modulates Fo47-induced response (FIR) against *V. dahliae*. HPLC measurements showed that pepper plants treated with Fo47 had increased levels of 12-oxo-phytodienoic acid (OPDA), salicylic acid, and jasmonyl isoleucine [10]. It is possible that there is no master hormone controlling FIR, but rather a crosstalk network that can buffer the lack of some components. Note that inhibiting the ethylene signalling either in pepper or tomato produces a partial loss or gain of the response, indicating parallel signaling pathways and/or summative antagonistic effects. The crosstalk among hormones in tomato has been observed several times. O’Donnell [34] demonstrated that tomato plants infected with *Xanthomonas campestris* pv. *vesicatoria* accumulate SA in correlation with necrosis, but in ethylene-insensitive plants, SA accumulation does not occur and necrosis is reduced. Exogenous addition of SA to ethylene-deficient tomato mutants restores necrosis, indicating that reduced disease symptoms are associated with failure to accumulate SA. Same as tomato, *Arabidopsis* also accumulates SA and ET after *X. campestris* inoculation, SA being responsible for the necrosis [35]. However, in *Arabidopsis*, SA accumulation is up-stream of ET accumulation, that is, the *Arabidopsis* NahG line does not accumulate SA or ET after pathogen infection, while ET-insensitive lines accumulate both SA and ET [35]. Tomato ET-insensitive, Never-ripe, does not accumulate SA after *X. campestris* inoculation [34]. Further investigation into *Arabidopsis* revealed that JA and auxin also accumulate after pathogen infection, but this accumulation was independent of SA or ET [35]. ET has been observed to have opposite effects on the susceptibility of tomato to *V. dahliae*. As observed in this work, Fo47 was able to protect the ET-insensitive tomato against *V. dahliae* (Figure 7), but it is known that ET application protects tomato from the related pathogen, *V. longisporum* [36]. This ET dichotomy has also been observed in tomato plants treated with the ET precursor, 1-aminocyclo-propane-1-carboxylate (ACC), at the time of inoculation to produce an initial transient burst of ET, and then blocked the ET production by adding the ethylene biosynthesis inhibitor aminoethoxyvinylglycine (AVG). By this consecutive treatment with ACC and AVG, symptom severity caused by *V. ahlia* is reduced further than each treatment alone [17]. Besides its involvement in resistance, ET controls symptom development in tomato [37]. Never-ripe tomato mutant exhibits a reduction of symptoms caused by *X. campestris*, but this reduction was not correlated with less development of the pathogen [37]. Moreover, the symptoms caused by *Verticillium* in tomato have been attributed to ET production since pretreatment with ET increased the symptomatology [38]. This ET- and pathogen-induced symptomatology has been related to gel formation that could occlude xylematic vessels [39]. Indeed, Never-ripe tomato showed reduced *V. dahliae* symptoms when compared with its control, showing 15% less reduction in fresh and dry weight and 23% less reduction in the stem length than Pearson plants. This reduction of symptomatology was also observed by Pantelides [40] in tomato when inoculated with *V. dahliae*. The importance of ET in symptom development has been demonstrated by Robison et al. [41]. In a tomato line unable to accumulate ET, the symptoms produced by *V. dahliae* were reduced, but not the pathogen presence in the plant tissues, suggesting that reduced ethylene synthesis results in increased disease tolerance [41]. Besides ET, JA is also related to *V. dahlia* resistance in tomato. Tomato mutants of the JA-signalling pathway, *defenseless-1* (*def1*), are more susceptible to *V. dahliae*; hence, tomato basal resistance against this vascular pathogen should involve JA-signaling [42]. The linoleic acid metabolism (Figure 5), which contains the lipooxygenase pathway or LOX pathway, was up-regulated in tomato after Fo47 treatment. However, the allene oxide synthase (AOS) branch of the LOX pathway was down-regulated, while the branch of the hydroperoxy lyase (HPL) was up-regulated (Figure 5 and Table 5). The HPL branch is responsible for the biosynthesis of volatile aldehydes and phytooxylipin traumatic acid. These are components of the response to wounding that are activated in response to mechanical wounding, but they have been also observed in tomato’s response to colonization by the beneficial mycorrhizal fungi *Rhizophagus irregularis* [43].

Moreover, genes in the response to auxin stimulus were observed in both plants, and auxin mediated signaling pathway genes have also been differentially expressed in pepper (Table 4). Root-interacting beneficial fungi *Piriformospora indica* and *Mortierella hyaline* induced auxin-responsive genes in the roots of their host [44]. An intricate crosstalk between these hormones seems to take place in the responses triggered in both pepper and tomato.

FIR (Fo47-induced response) activates several components of the defence response in both organisms, among them, several PR-proteins (Table 2). FIR acts partially through priming [6], but also leads to the direct activation of PR-genes. Activation of PR proteins by Fo47 has been observed at early steps of Fo47 colonization, 2 to 4 days, in pepper and tomato [6,7,45]. The tomato *PR-P6* gene is a pathogenesis-related protein induced by SA [46,47]. Even though, classically, PR1 has been classified as an SA marker in Arabidopsis [48], in other plants, such as pepper, it does not have a clear-cut response pattern, as it is activated by SA and ET [18].

Besides components related to the SA, JA, and ET pathways, auxins also take part in balancing the FIR. Both Solanaceae showed auxin related DE genes, especially in pepper (Table 4). In pepper, two Aux/IAAs were down-regulated and an ARF was up-regulated. Aux/IAAs are short-lived nuclear proteins, which inhibit auxin-response transcription factors (ARFs) [49]. Auxin is able to modulate JA response through interaction with repressors of the JA signaling pathway [50]. Expression of some ARFs has been demonstrated to induce JA production [51], and induction with MeJA can also increase auxin levels [52]. Auxin is able to induce JAZ proteins and therefore inhibit JA-signaling [50]. Hence, auxin can induce JA synthesis, but can also block its signaling by inducing its repressors. Priming is an important component of FIR [7] that has not been considered in this work. Priming is an enhanced response, only triggered after pathogen recognition that rewires the signaling response. Such response might include other components not observed here that need to be studied in future assays.

In conclusion, we have observed that Fo47 protects pepper cv Padron against *V. dahliae*, but not tomato cv Pearson. Inhibition of ET signaling in pepper reduces the Fo47 protection, while in tomato, this protection is enhanced in the ET-insensitive mutant Never-ripe. Other hormones such as oxylipins and auxins might have a role in FIR that needs to be tested in future assays.

## Figures and Tables

**Table 1:** Genes differentially expressed in both pepper and tomato after Fo47 induction.

Gene ID	Gene	Fold	Description
107864567 778321	STH2	2.57 (Pepper) 6.24 (Tomato)	Pathogenesis-related protein STH-2-like
107840155 100191111	PR1	3.41 (Pepper) 4.42 (Tomato)	Basic form of pathogenesis-related protein 1-like
107877508 101245668	XET24	0.23 (Pepper) 0.30 (Tomato)	Xyloglucan endotransglucosylase/hydrolase protein 24-like
107873721 544295	Dif54	0.24 (Pepper) 0.10 (Tomato)	Extensin-like protein

**Table 2:** Pathogenesis related genes differentially expressed in pepper and tomato after Fo47 treatment.

Gene ID	Gene	Organism	Fold	Description
544083	PR-1a1	Tomato	2.27	Solanum lycopersicum PR-1a1
107864567778321	STH2	Pepper Tomato	2.57 6.24	Pathogenesis-related protein STH-2-like
544069	PR-P2	Tomato	2.14	Solanum lycopersicum PR-P2
107840155 100191111	PR1	Pepper Tomato	3.41 4.42	Basic form of pathogenesis-related protein 1
107842907	P6	Pepper	2.25	Pathogenesis-related protein P6

**Table 3:** Genes differentially expressed for the ethylene biosynthesis and signaling pathway in pepper and tomato after Fo47 treatment.

Gene ID	Gene	Organism	Fold	Description
107839239	ACS2	Pepper	3.39	1-aminocyclopropane-1-carboxylate synthase
107860267	ERF011	Pepper	2.97	Ethylene-responsive transcription factor
107853805	ACO1	Pepper	2.73	1-aminocyclopropane-1-carboxylate oxidase
107872603	ERF-1like	Pepper	0.33	Ethylene-responsive transcription factor
107865816	ERF-T	Pepper	0.32	Ethylene-responsive transcription factor
107866610	ERF096	Pepper	0.26	Ethylene-responsive transcription factor
543800	ACO5	Tomato	3.40	1-aminocyclopropane-1-carboxylate oxidase
101253257	ERF017	Tomato	0.34	Ethylene-responsive transcription factor

**Table 4:** Genes differentially expressed for the response to auxin stimulus in pepper and tomato after Fo47 treatment.

Gene ID	Gene	Organism	Fold	Description
778363	ARF3	Pepper	3.09	Auxin response factor 3
101256828	SAUR50	Pepper	2.11	SAUR-like auxin-responsive protein
101055544	IAA5	Pepper	0.46	Auxin-responsive protein
101055549	IAA19	Pepper	0.31	Auxin-responsive protein
101251823	KRP1	Tomato	0.46	Calcium-binding protein KRP1
101265243	SAUR71	Tomato	0.50	SAUR-like auxin-responsive protein
101259898	BT4	Tomato	0.48	BTB and TAZ domain protein 4

**Table 5:** Genes differentially expressed for the α-Linolenic acid metabolism in tomato after Fo47 treatment. KEGG pathway (SLY00592) with a *p*-value of 3.7 × 10^−2^.

Gene ID	EC	Gene	Organism	Fold	Description
544008	1.13.11.12	loxC	Tomato	2.31	Lipoxygenase
543642	HPL1	HPL1	Tomato	2.09	Fatty acid hydroperoxide lyase
606711	4.2.1.92	AOS	Tomato	0.28	Allene oxide synthase

**Table 6:** Genes differentially expressed for the cysteine and methionine metabolism in pepper and tomato after Fo47 treatment. KEGG pathway (CANN00270) with a *p*-value of 4.4 × 10^−2^.

Gene ID	EC	Gene	Organism	Fold	Description
107879648	2.7.2.4 1.1.1.3	AK-HSDH1	Pepper	3.52	Bifunctional aspartokinase/homoserine dehydrogenase 1
107839239	4.4.1.14	ACS2	Pepper	3.39	1-aminocyclopropane-1-carboxylate synthase
107853805	1.14.17.4	ACO1	Pepper	2.73	1-aminocyclopropane-1-carboxylate oxidase
543800	1.14.17.4	ACO5	Tomato	2.97	1-aminocyclopropane-1-carboxylate oxidase

## Data Availability

Microarray data has been deposited in the NCBI Gene Expression Omnibus (GEO) repository. The accession number is GSE49432.
